# Heparin and Derivatives for Advanced Cell Therapies

**DOI:** 10.3390/ijms222112041

**Published:** 2021-11-07

**Authors:** Sandra Laner-Plamberger, Michaela Oeller, Eva Rohde, Katharina Schallmoser, Dirk Strunk

**Affiliations:** 1 Department of Transfusion Medicine, Paracelsus Medical University of Salzburg, 5020 Salzburg, Austria; s.laner-plamberger@salk.at (S.L.-P.); m.oeller@salk.at (M.O.); e.rohde@salk.at (E.R.); 2 Spinal Cord Injury and Tissue Regeneration Center Salzburg, Paracelsus Medical University of Salzburg, 5020 Salzburg, Austria; dirk.strunk@pmu.ac.at; 3 Cell Therapy Institute, Paracelsus Medical University of Salzburg, 5020 Salzburg, Austria

**Keywords:** heparin, cell therapy, regenerative medicine, biomaterials, human platelet lysate, extracellular vesicles

## Abstract

Heparin and its derivatives are saving thousands of human lives annually, by successfully preventing and treating thromboembolic events. Although the mode of action during anticoagulation is well studied, their influence on cell behavior is not fully understood as is the risk of bleeding and other side effects. New applications in regenerative medicine have evolved supporting production of cell-based therapeutics or as a substrate for creating functionalized matrices in biotechnology. The currently resurgent interest in heparins is related to the expected combined anti-inflammatory, anti-thrombotic and anti-viral action against COVID-19. Based on a concise summary of key biochemical and clinical data, this review summarizes the impact for manufacturing and application of cell therapeutics and highlights the need for discriminating the different heparins.

## 1. Introduction

The discovery of heparin more than one hundred years ago happened as an unexpected coincidence. At the beginning of the 20th century, William Howell and co-workers were studying a pro-coagulant ‘thromboplastic substance’ [1] consisting of phospholipids and tissue factor. Literally, investigating pro-coagulant phospholipids from different tissues, Howell’s student Jay McLean observed an anticoagulant effect of ‘cuorin’ and ‘heparphosphatid’, preparations isolated from heart and liver extracts in 1916 [2]. In 1918, Howell and Holt identified ‘heparin’ as a novel anti-coagulant [3]. Nowadays, these historical scientific results are difficult to interpret, because analyzed ‘substances’ were mainly complex compounds with unavoidable impurities. As the first experiments on animals were promising, heparin turned out to be a potent anticoagulant drug in the following decades. In 1939 and 1942, the FDA approved bovine and porcine heparin, respectively. In parallel, the awareness for the clinical significance of heparin increased in Europe and commercial production started in Switzerland in 1939. However, it took until the 1970s before the exact chemical composition and mode of action were uncovered (for review see [4]). In recent decades, low molecular weight heparins (LMWH) and oral anticoagulants have almost completely replaced unfractionated heparin (UFH) in clinical practice, mainly due to easier use and better safety profile at comparable efficiency for the vast majority of applications [5]. While clinical use of UFH as a highly potent medication is now restricted to a limited number of selected cases, e.g., [6,7,8], it saw a revival in cell manufacturing when using human platelet lysate instead of fetal bovine serum [9,10,11], and for application of cell-based medicinal products to avoid an instant blood-mediated inflammatory reaction (IBMIR) [12,13,14]. It is perhaps not common knowledge that heparins have clinical efficiency as anti-inflammatory [15,16], anti-cancer [17,18] and anti-viral therapeutics, especially in the new coronavirus (SARS CoV-2) pandemic [19,20], is not definitively clarified, and further clinical data are urgently needed. In regenerative medicine, the investigation of the advantages, disadvantages and adverse effects of UFH compared to LMWH, heparin pentasaccharides and also oral anticoagulants is also still pending.

## 2. Biology, Biochemistry and Clinical Application

### 2.1. Heparin Structure and Anticoagulant Mode of Action

Heparin is a linear, unbranched and highly sulfated polysaccharide belonging to the family of glycosaminoglycans (GAGs) [21,22]. Based on various repeating disaccharide units, four main groups of GAGs can be distinguished: (i) heparin and heparan sulfate, (ii) chondroitin sulfate and dermatan sulfate, (iii) keratan sulfate, and (iv) hyaluronan. The repeating disaccharide units of heparin consist of uronic acid and D-glucosamine connected by α-glycosidic linkage. Membrane-bound heparan sulfate proteoglycans (HSPGs) include syndecans-1–4, glypicans 1–6, betaglycan, neuropilin-1 and CD44. Serglycin on secretory vesicles and perlecan, agrin and collagen XVIII within extracellular matrix complete the HSPG family [23]. In vivo, heparin is mainly synthesized and stored by mast cells in their secretory granules [24]. The knockout of glucosaminyl N-deacetylase/N-sulphotransferase-2 (NDST-2), which is important for initial modification steps during heparin biosynthesis, completely abrogated heparin synthesis in murine mast cells. However, despite alteration of mast cell morphology and granules, NDST-2 knockout mice were viable and fertile. As no thrombotic events were observed, endogenous heparin may not be indispensable for the regulation of blood coagulation [25,26].

**Figure 1:**
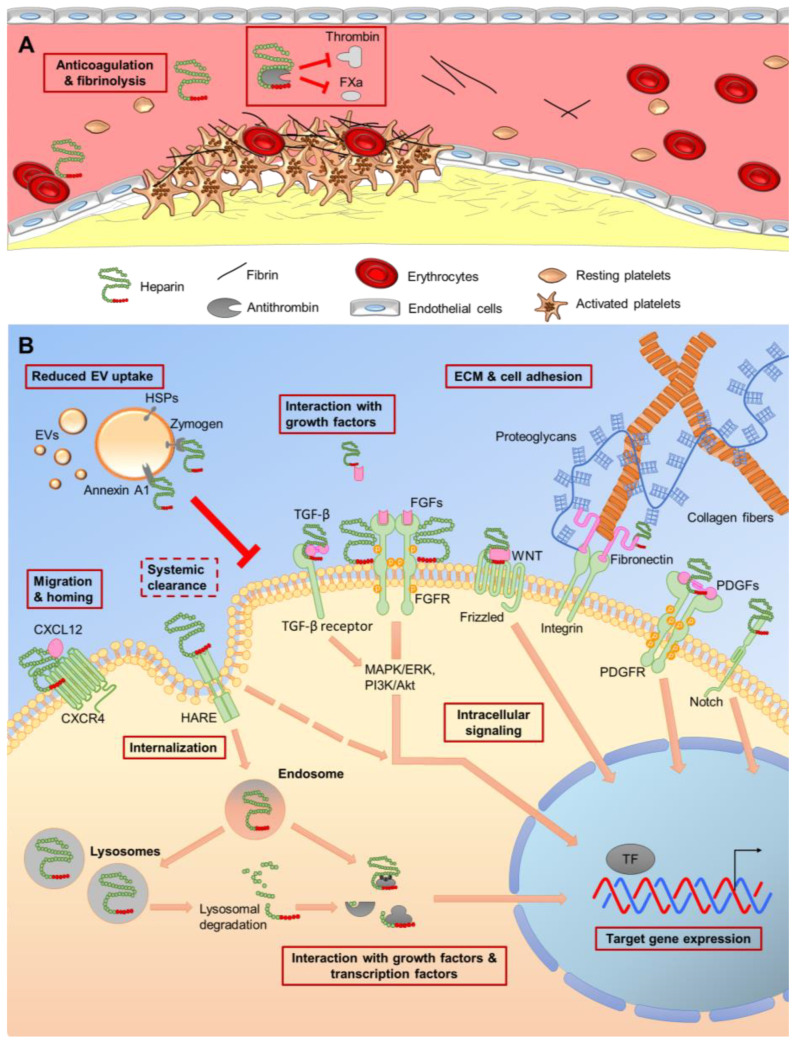
Overview of intravascular, extracellular and intracellular effects of heparin. (**A**) In the blood stream, the anticoagulant effect of heparin results from binding to antithrombin amplifying inhibition of activated factor Xa (FXa) and thrombin. (**B**) In the extracellular and intracellular environment, heparin affects essential cell functions such as ECM formation, cell adhesion and migration. The interaction with the extracellular matrix (ECM), but also growth factor receptors as transforming growth factor (TGF)-beta receptor, fibroblast growth factor receptor (FGFR) Frizzled, Notch and platelet derived growth factor receptor (PDGFR) activates divergent intracellular signaling pathways, putatively affecting gene expression. A systemic clearance of heparin by cellular internalization and lysosomal degradation may finally induce the expression of target genes. Furthermore, the cellular uptake of extracellular vesicles (EVs) can be reduced by heparin, putatively influencing cell-to-cell communication.

The anticoagulant effect of heparins is in part mediated by a unique pentasaccharide sequence binding with high affinity to antithrombin [27]. This induces a conformational change in its reactive center leading to an up to 1000-fold enhanced inactivation rate of thrombin and activated coagulation factor X (FXa) by antithrombin [28]. For therapeutic purposes, three forms of heparins are available: (i) UFH, (ii) LMWH and (iii) synthetic ultra (U)LMWH, the latter corresponding to just five to ten saccharide molecules [29] (Table 1 and Figure 1A). 

Commercially available UFH is isolated from porcine intestinal mucosa or lung and intestine from cattle, the only sources for industrial production. As outlined recently [29], the complex manufacturing process of pharmaceutical-grade heparins includes the purification, isolation and drying of highly charged heparin molecules from other GAGs, with details of the production methodology usually kept secret. Due to animal origin and biosynthesis, GAG molecules are of highly variant chain lengths and sulfation patterns, finally impeding the perfect purification of UFH. This became fatal reality in 2007 and 2008 in the ‘heparin contamination crisis’. Structurally related over-sulfated chondroitin sulfate impurified an adulterant heparin product and was not detected by standard quality control. This caused hundreds of anaphylactic reactions and several deaths worldwide [30], fostering the need for synthetic heparins and alternative anticoagulants.

UFH (average molecular weight of 19,000 Da) has several advantages including short onset of action, no placenta passage, feasibility for patients with renal failure, neutralization by protamine and low price. Nevertheless, the limitations predominate, particularly the need for parenteral administration and dose monitoring, due to variable bioavailability. In clinical practice, the individual dose is adjusted, e.g., six-hourly, usually according to the activated partial thromboplastin time (aPTT) and the anti-FXa activity, both poorly reflecting the anticoagulant effect [4]. As UFH inhibits the thrombin forming capacity of plasma mainly by its anti-thrombin activity and minor by inhibition of FXa, the analysis of the endogenous thrombin potential reflects the anticoagulant effect more precisely. Particularly due to its extreme variability in the population but stability in the individual, the thrombin generation capacity is an important predictor of thrombotic risk and anticoagulant therapy [4].

LMWHs are produced from UFH by chemical and enzymatic depolymerisation, yielding smaller polysaccharide fragments (12–22 monosaccharide units with an average molecular weight of 5000 Da). Compared to an anti-FXa/anti-thrombin activity ratio of one for UFH, for LMWHs this ratio is between two and five [29]. Due to low affinity for plasma proteins, endothelial and blood cells, LMWHs show better subcutaneous bioavailability and longer half-life (3–6 h) enabling application once or twice daily without the need for laboratory monitoring. Causing fewer adverse reactions than UFH, LMWHs have been recommended for prophylaxis and therapy of thromboembolic events since the 1990s [34,35]. 

As just one third of individual heparin molecules show anticoagulant properties, further attempts have been made to biochemically synthesize only ULMWHs or pentasaccharides, as fondaparinux (Arixtra^®^) being the first and only synthetic clinically approved selective FXa-inhibitor [29]. In sum, LMWHs and synthetic pentasaccharides have a greater capacity to accelerate the inhibition of FXa than the inhibition of thrombin [35,36]. 

### 2.2. Clinical Application of Heparins in Infections, Inflammation and Cancer

The most important clinical application of LMWH is prophylaxis and treatment of deep vein thrombosis, stroke and pulmonary embolism in medical and surgical patients [35,37]. Another cutting-edge indication for heparins is the coagulopathy of severely ill patients with acute respiratory distress syndrome (ARDS) due to the novel corona-virus disease 2019 (COVID-19) in the current pandemic [38]. Notably, in this inflammatory and pro-thrombotic state of COVID-19 infection, an elevation of the coagulation potential may require higher heparin doses than the standard dose, implicating a “functional” heparin resistance and again emphasizing the need for more reliable monitoring than anti-FXa activity by thrombin generation analysis [39]. 

The benefits of prophylactic or therapeutic UFH or LMWH for patients with thromboembolic events and sepsis-induced disseminated intravascular coagulation, concomitant with high D-dimer and fibrinogen and low anti-thrombin levels, are currently discussed and investigated in clinical trials [19,38,40]. In a recent multicenter randomized clinical trial (HEP-COVID) a clear benefit of therapeutic-dose LMWH (with enoxaparin, major thromboembolism or death in 28.7%) compared to prophylactic or intermediate-dose heparin regimens (with UFH, enoxaparin or dalteparin, major thromboembolism or death in 41.9%) in inpatients with high D-dimer levels has been reported [41]. Preliminary data from a large multiplatform of randomized controlled trials (ATTACC, REMAP-CAP and ACTIV-4a trial platforms), including more than 2000 patients comparing therapeutic LMWH or UFH to local venous thromboembolic prophylaxis in severely and moderately ill COVID-19 patients, showed divergent results [39]. There was a benefit from therapeutic anticoagulation in the moderate disease severity group, but the mortality in the total patient cohort was not significantly influenced by the heparins. Additionally, a comparison of prophylactic (40 mg enoxaparin) to intermediate (1 mg enoxaparin/kg body weight) LMWH in 562 patients with severe COVID-19 infection in another randomized clinical trial (INSPIRATION) showed no benefit from increasing the LMWH dose [42]. In a recent open-label multicenter randomized clinical trial (ACTION), 615 COVID-19 patients with elevated D-dimer levels received either therapeutic or prophylactic anticoagulation. In the therapeutic group, stable patients were treated with oral rivaroxaban; unstable patients were treated with enoxaparin or UFH followed by rivaroxaban. The prophylactic group received subcutaneous standard dose of enoxaparin or UFH. The primary efficacy outcome (time to death, duration of hospitalization or duration of oxygen supplementation) was not different between the groups but bleeding complications were increased by therapeutic anticoagulation with rivaroxaban [43]. 

Based on these preliminary data, a general benefit of heparins compared to no heparins seems evident only in selected COVID-19 patients with reduced disease severity. There was no further improvement observed with higher doses of heparins, but instead increased risk of bleeding complications, especially when combined with direct FXa inhibitors. Therefore, for clinical practice, several comprehensive guidelines about prophylaxis and therapy of thromboembolic complications in COVID-19 infection have been published in the last months, e.g., by the British National Institute for Health and Care Excellence and the American Society of Hematology [44,45], and are highly recommended. 

In COVID-19 disease, not only anticoagulant, but also anti-inflammatory and anti-viral effects of heparin and derivatives may be beneficial, as UFH and heparin derivatives have been supposed to inhibit viral and protozoan infections by impeding the interaction between pathogen proteins and heparan sulfate chains on the cell surface [46]. Pathogen proteins being responsible for cell entry such as HIV-1 gp120 [47,48], Dengue virus envelope protein [49], and circumsporozoite protein, a cell surface protein of the parasite plasmodium falciparum causing malaria [50], were shown to interact with UFH and heparin derivatives. Furthermore, the interaction of Clostridium difficile toxin A with de-N-sulfated heparin inhibited the cell entry, in contrast to highly O-sulfated heparins enhancing it [51]. A recent study, however, showed that UFH and LMWH inhibited Dengue virus but promoted Zika virus replication [52]. These results indicate that UFH and LMWH molecules may act either pro- or anti-pathogenic, depending on various sulfation patterns and in a context-dependent manner. 

UFH and LMWHs may have an anti-inflammatory potential [53]. Over the last years, treatment of various inflammation-associated diseases (e.g., bronchial asthma, rheumatoid arthritis, cystic fibrosis and inflammatory bowel disease) with UFH and LMWHs was evaluated in clinical trials, with conflicting results [15,53,54]. As analyzed in a systematic review [15], patients with active ulcerative colitis had no benefit from LMWH by injection but only from LMWH administered in high dose by extended colon-release capsules. 

In cancer patients, the incidence of arterial and venous thromboembolism is increased, frequently causing morbidity and death. These thromboembolic events are triggered by various clinical risk factors such as surgery, immobilization, type and stage of the primary tumor, hormone- and chemotherapy [55,56,57]. This creates the need for consequent thromboprophylaxis with LMWHs, with UFH in patients with coexisting renal failure, and with direct oral anticoagulants (DOACs) [58,59]. In randomized clinical trials, DOACs were found to be similarly effective as LMWHs but had a higher risk of bleeding, particularly in patients with thrombocytopenia, intracranial and hematological malignancies or due to drug-drug interactions [59].

The anticoagulant effect of UFH and LMWHs is not only induced by inactivation of plasmatic coagulation factors, but also by reduced platelet activation via protease-activated receptor 1 (PAR1), due to diminished thrombin formation. Platelets are essential contributors of cancer-associated thromboembolism but can also nurture tumor growth and metastasis, as reviewed comprehensively [60]. These complex interactions are characterized by cancer-associated thrombocytosis [57], protection of tumor cells from apoptosis and NK cell attack by platelet shielding and transfer of unaffected major histocompatibility class I molecules onto the tumor cell surface [60]. Furthermore, platelets store a plethora of growth factors and cytokines in their specific granules [10], supporting angiogenesis and tissue repair not only in wound healing. These mediators are released after platelet activation and also play a pivotal role in tumor growth and metastasis [60,61]. P-selectin, for example, is stored in platelet alpha-granules under steady state conditions and gets expressed on the platelet surface after activation. Evidence exists indicating that binding of platelets to tumor cells may depend on P-selectin contributing to microembolic events and metastasis [60]. Notably, differential inhibitory effects of UFH and LMWHs on platelet aggregation [62] and on selectins [63,64] have already been shown some decades ago, indicating the non-anticoagulant effects of heparins in cancer. 

Further potential non-anticoagulant anti-cancer effects of heparin were already described in 1957, demonstrating an inhibitory effect of UFH on ascites tumors in mice [65]. Furthermore, experimental animals did benefit from UFH and LMWHs by reduced tumor growth and diminished metastasis [53,66]. Later animal studies reproduced that UFH decreased tumor cell adhesion, and that LMWHs diminished metastasis burden and primary tumor growth in animal cancer models, but the overall survival of solid tumor patients was not increased by LMWHs [17,67]. Due to the limitations as heterogeneity of number, dosing and timing of treatment, more standardized study protocols and investigation of the exact dose-response relationship would be required for exactly predicting clinical effects of LMWHs during anti-neoplastic therapy [67]. As anti-inflammatory and anti-cancer effects of heparin and derivatives were mainly observed with high doses, the concomitant risk of bleeding complications may hinder efficient therapy [16]. Highly sulfated synthetic or semi-synthetic heparin mimetics with reduced anticoagulant activity have been developed to overcome this problem [68]. 

### 2.3. Adverse Effects of Heparin Treatment

The most common adverse effect of heparins is bleeding. The incidence of major bleeding complications ranged from 2% with LMWHs to more than 5% with intravenous UFH [69]. The individual risk depends on the dose, surgical technique, underlying disease and concomitant medication, e.g., platelet aggregation inhibitors or cytostatic agents [33]. Algorithms for the management of this iatrogenic hemorrhagic diathesis have been developed [69], and protamine can be employed as specific antagonist for UFH and LMWH. 

A frequently observed transient and mild decrease in platelets due to a nonimmune-mediated effect of heparins is termed type I heparin-induced thrombocytopenia (HIT). The massive platelet drop on days 3 to 5 of heparin therapy is a rare but potentially life-threatening side effect. The characteristic symptoms of a more distinct thrombocytopenia and arterial embolism, now considered type II HIT, were first described by Weismann and Tobin in 1958 [70]. The mechanism of this adverse drug reaction was extensively studied over the past decades [71]. The causative agents are mainly IgG antibodies against complexes of heparin and platelet factor 4 (PF4) binding to the immunoglobulin Fcɣ receptor IIa on platelets, with the potential to induce platelet activation and consumption, consecutive thrombin generation and paradoxical thromboembolic events [71]. The incidence for HIT II ranges from 0.1% to 7% of patients exposed to heparin, depending on UFH or LMWH type, the underlying disease, surgical interventions and other factors [72]. Further details about HIT diagnosis, the management of HIT-associated thromboembolic events and treatment with alternative anticoagulants are out of the scope of this review, and are summarized in recent comprehensive guidelines of the American Society of Hematology [72]. Other observed adverse effects of heparin treatment are osteoporosis, skin lesions, alopecia and the elevation of hepatic enzymes [73].

### 2.4. Modulation of Extracellular Matrix and Cell Adhesion

A variety of stimuli regulates the extracellular matrix (ECM) conformation, including mechanical forces and different ligands [74]. During ECM assembly, fibronectin fibrils interact with collagens, proteins and growth factors to build the final matrix. Fibronectin has binding sites for heparan sulfate and heparin, influencing fibronectin conformation and regulating growth factor presentation at the cell surface [75,76]. UFH and LMWHs may differentially influence cell adhesion via neural cell adhesion molecule 1 (NCAM1, CD56) [77], selectins and integrins [63,64,78,79]. Indirectly, via macrophage receptor 1 (Mac-1, CD11b/CD18) inhibition, UFH and LMWH reduced leukocyte adhesion on endothelial cells via intercellular adhesion molecule 1 (ICAM-1, CD54) [80], to cite just selected effects. Depending on conformational changes influencing ECM properties heparin binding can thus increase or decrease adhesion. Heparin molecules as GAGs can impact biological processes by specific interaction with growth factors, cytokines and chemokines, cell adhesion molecules, and cell surface proteins of pathogens [16,46,54,68], depending on dose, the saccharide chain length, specific orientation and arrangement of its sulfo- and carboxyl-groups. This makes heparins important multifunctional mediators in cell signaling and gene expression, influencing cell fate beyond coagulation (Figure 1B).

### 2.5. Gene Expression Modulation

Heparin and heparan sulfate significantly regulated genes involved in cell adhesion and proliferation in human bone marrow-derived stromal cells in a donor-dependent manner [81,82]. UFH also regulated gene expression, depending on the tissue source of stromal cells at a therapeutic dose of 2 IU/mL [83]. Independent of the cell source and concentration of UFH, mainly genes affecting cell proliferation (e.g., members of the WNT-, PDGF- and Notch signaling pathways), adhesion, apoptosis and angiogenesis were upregulated. Downregulated genes were involved in inflammatory processes, cytokine and chemokine signaling and negative regulation of WNT-, TGFβ- and EGFR-pathways. It is still not completely understood how heparin affects gene expression precisely, but there are at least two explanations: (i) UFH can bind to cell surface receptors, support their activation, as observed for fibroblast growth factor receptors, FGFRs [84,85], thereby fostering intracellular signaling, leading to modified gene expression pattern. (ii) It was also shown to be internalized and directly interfere with transcription factors [86]. The systemic clearance of heparins from the circulation was found to be tightly linked with the hyaluronan receptor for endocytosis (HARE/stabilin-2) [87,88,89] (Figure 1B). 

### 2.6. Effect on Cell Proliferation and Differentiation

The effect of UFH supporting long-term propagation of endothelial cells is well-known [90]. It plays a pivotal role in cell proliferation acting as co-factor for growth factors of the FGF family [84,91], the transforming growth factor (TGF)-beta superfamily [92,93], vascular endothelial growth factors (VEGFs) [94,95], placental growth factor (PlGF) [96,97] and platelet derived growth factors (PDGFs) [98], among others. Members of the FGF family were shown to require an interaction with the corresponding high affinity receptor and heparins to realize their full signaling potential [99,100,101]. Heparins also interacted with other mitogenic factors such as midkine (previously named neurite growth promoting factor 2, NEGF2) [102] and hepatocyte growth factor (HGF) [103]. These interactions supposedly induce structural changes, stabilizing the tertiary structure of the growth factors and resulting in a potentiated growth promoting activity [104]. High-resolution x-ray studies revealed that heparin and heparan sulfate bound to FGFs and promoted the dimerization of FGFRs, thus inducing FGF signal transduction [84,85]. UFH further protected FGF from proteolytic cleavage [105] and basic or acidic inactivation [106] and increased the diffusion radius by influencing the binding of the growth factors to ECM proteoglycans [107]. 

The effect of heparins on cell proliferation in vitro can be either growth promoting or inhibiting. It appeared to be mandatory for efficient proliferation of endothelial cells and their progenitors [108,109]. Stromal cell growth was also observed to be only partly stimulated by UFH, strongly dependent on their tissue origin [83,110]. Low concentrations of UFH supported proliferation of human bone marrow-derived stromal cells and human embryonic stem cells; higher concentrations impaired cell growth in a dose-dependent manner [111,112]. The cell type and dose-dependent variability of heparin’s effects on proliferation is reminiscent of the adhesion-increasing or -decreasing effects discussed above. 

GAGs are also tightly linked to developmental and differentiation processes. In animal models, proteoglycans were identified as important modulators of protein gradient formation and signal transduction [113,114]. UFH was demonstrated to promote the osteogenic differentiation of human bone marrow stromal cells in vitro [115,116,117]. The effect on osteogenic differentiation was tightly linked to the sulfation pattern. While UFH and 2-O-desulfated heparin stimulated osteoclastogenesis, N-desulfated heparin exerted suppressive effects on osteoclastogenesis and bone resorption in vitro and in vivo [118,119]. Historical [120] and more recent clinical research [121] however demonstrated adversely effected bone density in up-to one third of heparin-treated patients, particularly after extended exposure [122].

## 3. Heparins for Manufacturing Cell Therapeutics 

### 3.1. Heparins as a Cell Culture Supplement

Endothelial cell culture as a prerequisite for studying molecular mechanisms of vascular biology and regeneration was established half a century ago [90,123]. Addition of UFH to culture medium containing reduced concentrations of endothelial cell growth factor enabled cloned human endothelial cell strain propagation for the first time in the early 1980s [109]. In recent years, novel UFH applications appeared, especially in the field of cell-based therapeutics, making UFH a key component in subsequent clinical-grade manufacturing of endothelial and stromal cells [124,125]. As the European Medicines Agency recommended the avoidance of animal-derived components for manufacturing cell therapeutics [126], human platelet lysate (HPL) has been implemented as an efficient cell culture supplement [9,127,128,129,130,131]. HPL supports in vitro cell proliferation due to abundant growth factors and cytokines superior to fetal bovine serum (FBS) [10,132,133,134]. Because HPL contains fibrinogen and plasmatic coagulation factors, addition of ideally preservative-free UFH to the HPL-supplemented culture medium is mandatory to avoid jellification-like clotting events during cell propagation [112]. Although UFH is of porcine origin, there are still no alternative anticoagulants of human origin established for cell culture. Recombinant serglycin decorated with heparin/heparin sulfate represents one strategy to replace UFH in cell culture [135]. Recombinant hirudin derivatives and non-heparin synthetic anticoagulants are efficiently used to treat patients with heparin-induced thrombocytopenia type II [136], but may contain preservatives and are still not validated as cell culture additive. Notably, effects of heparins on cell biology as described above in detail can be considered operative also in cell culture. 

### 3.2. Potential Benefits of Heparins for Cell Therapy

Solid organs and different progenitor cell types can be transplanted successfully due to human leukocyte antigen matching and pharmacologic immune suppression strategies. After liver cell transplantation, an initially unexplained substantial cell loss after application was discovered through attentive observation [137,138]. The instant blood-mediated inflammatory reaction (IBMIR) [139], an innate immune attack characterized by the activation of the complement system and coagulation cascade, was shown to be a main cause of the substantial cell loss after extra-hematopoietic cell transplantation, particularly of isolated human hepatocytes and Langerhans’ islets. During IBMIR, binding of activated platelets to the transplanted cells and consecutive clot infiltration by neutrophil granulocytes and monocytes, is eventually leading to cell destruction [137,140]. In different studies it was demonstrated, that most types of culture-expanded stromal cells, with the exception of bone marrow-derived stromal cells lacking tissue factor (coagulation factor 3, FIII), also trigger significant clotting events in vitro as well as in vivo [13,141,142,143]. It was therefore suggested, that IBMIR is involved in the early cell loss and lack of engraftment after transplantation [144,145]. The majority of transplanted cells was shown to be trapped in thrombi in lung, liver and kidneys of transplanted animals [141,142,145,146,147] and human patients [148,149,150]. Clinical trials comparing efficiency and safety of UFH, LMWH, pentasaccharides or oral anticoagulants for IBMIR prophylaxis are still missing.

In order to prevent thrombotic complications after transfusion, several animal studies and subsequent clinical trials were efficiently using UFH as a pretreatment [140,151,152] during the preparation of, or directly combined with cellular therapeutics [12,153,154]. Further efforts to avoid necessity of systemic anticoagulation, which associates with a measurable bleeding risk, were based on results from efficient pancreatic islet surface heparinization [151]. On the surface of stromal cells and hepatocytes, heparin conjugates (consisting of about 70 heparin molecules of 13 kDa covalently bound to a polyamine chain with disulfide bonds) were immobilized by binding of polyethylene glycol-conjugated phospholipid (PEG-lipid) derivatives to a short heparin-binding peptide [155] to protect the cells from IBMIR-induced damages. This conjugation technique was further improved using a conjugate of heparin-binding peptide and human serum albumin on the surface of endothelial cells [152]. These strategies might appear in contradiction to the above-mentioned avoidance of UFH in cases where SDF-1/CXCR4-dependent homing might be affected [156], well representing another example of pleiotropic and partly dose-dependent heparin effects to be considered in cell therapy and regenerative medicine. 

### 3.3. Heparins in Biomaterials Used for Regenerative Medicine

**Figure 2:**
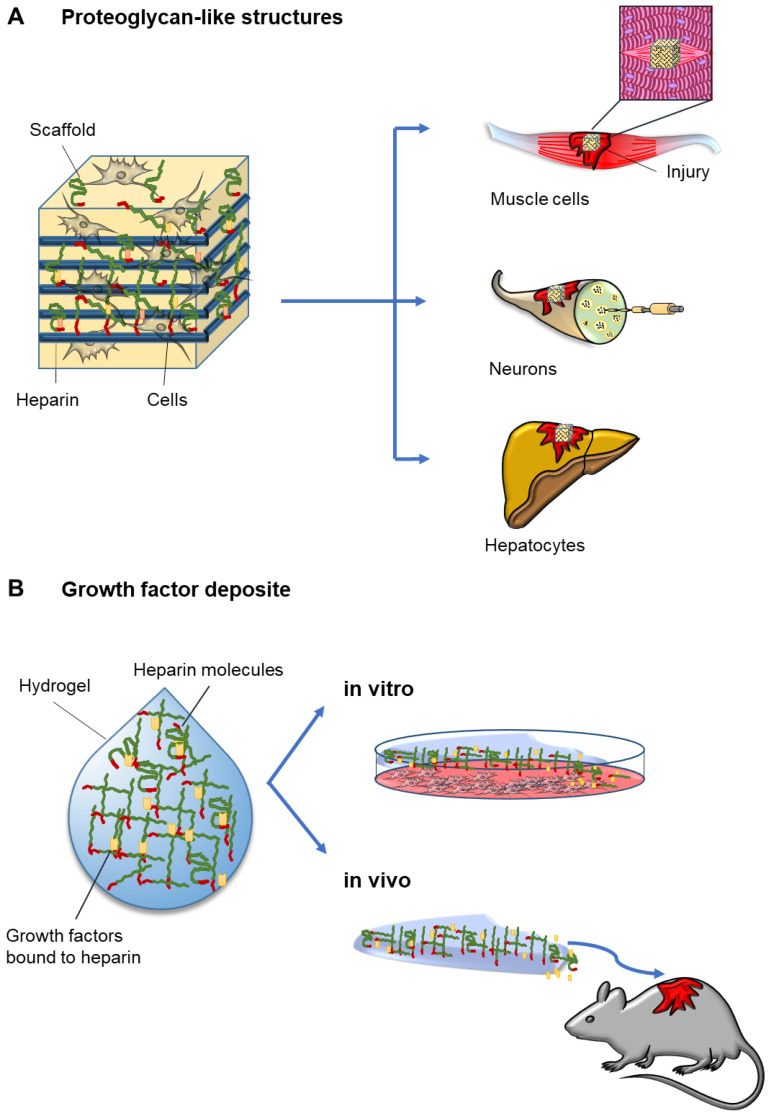
Heparin-based biomaterials for regenerative medicine. (**A**) Biodegradable scaffolds conjugated with heparin provide a proteoglycan-like structure. This mimics the physiologic functions of heparan sulfate, supporting proliferation and differentiation of muscle cells, neurons and hepatocytes. (**B**) In heparin-modified hydrogels and scaffolds, heparin molecules are frequently conjugated with growth factors and/or cytokines. These factors are slowly but constantly released during the degradation of the biomaterial and thus support cell proliferation and differentiation in vitro and in vivo.

A growing number of biotechnology applications is using heparins to support the production of specific cell-based therapeutics. The precise nature of the heparins (UFH or LMWH) was commonly not disclosed. The most frequently used heparin-based biomaterials include heparin-functionalized surfaces (either by electrostatic interactions, self-assembly or chemically immobilized), heparin-based hydrogels (either physically or chemically crosslinked), and heparin-containing nanoparticles, micelles or so-called coacervates, spontaneous aggregates of amphiphilic molecules [157]. Heparins are attractive components of biomaterials aiming to support different aspects of regeneration mainly for two reasons: First, biocompatible materials can be conjugated with heparin in order to provide a proteoglycan-like structure mimicking the physiologic functions of heparan sulfate. This was considered promoting proliferation and differentiation, for example of muscle progenitor cells [158,159,160,161], neurons [162,163], stromal cells [117] and hepatocytes [164,165] (Figure 2A). Second, UFH molecules were described to effectively bind a large number of growth factors and cytokines due to its high negative charge [104]. Gel-matrices or biodegradable scaffolds often contain intermediate size heparin molecules (>5000 Da) that interact with and therefore retain growth factors such as FGFs [166], VEGFs [167,168], PDGFs [169,170] or bone morphogenetic protein 2 (BMP-2) [171], frequently also in combination with SDF-1 [172], cytokines such as interleukin 10 (IL-10) [173], or other growth factors [174,175,176]. These heparin-interacting factors were shown to be released slowly and in a controlled manner from heparin-modified biocompatible scaffolds or hydrogels, thus providing stable local growth factor concentrations and therefore enhancing cellular growth and differentiation (Figure 2B). Heparin-containing matrices have been used in animal models to treat central nervous injuries [177], urinary incontinence [178], bone defects [179], skin wound healing [180] and to optimize the production of suitable replacements for corneas used in human eye surgery [181]. A multiplicity of in vitro and in vivo studies demonstrated that heparin is important not only as a cell culture supplement but also as a cell therapy adjuvant.

Heparins were successfully tested for tissue engineering (not covered in this review) and improving the production of cell therapeutics as a key component of diverse biomaterials. These functions, which are quite different to the anticoagulant properties of soluble heparin, might be explained in part also by the immobilization and associated modifications, which are supposed to affect heparin’s functionality in addition to the multiplicity of mechanistic explanations discussed in detail above.

## 4. Conclusions

A century after heparin discovery, mainly LMWHs and synthetic pentasaccharides are used in daily clinical practice together with oral anticoagulants to prevent and cure thromboembolic events [5]. However, UFH is far more than solely anticoagulant. Different heparins can act as modulators of key processes during cell adhesion, migration, communication, proliferation and differentiation in vitro and in vivo. The application of cell-based therapeutics often demands the in vitro use of UFH to support proliferation and/or differentiation of certain cell types, in addition to its increasingly common addition to HPL-based cell cultures. UFH and LMWH are also beneficial in vivo to prevent IBMIR and thromboembolic complications after transfusion of originally extravascular stromal cells expressing tissue factor. The impact of heparins on physiological processes seems to be mainly related to the interaction of cells with bioactive molecules. These interactions lead to a local concentration and furthermore induce conformational changes of proteins, thereby affecting the protein’s properties regarding target affinity and specificity. A more detailed understanding and more precise reporting of the different heparin’s influence on cell biology is mandatory for the future design of cell-based therapies particularly regarding the bleeding risk associated with UFH use.

## Figures and Tables

**Table 1:** Characteristics of unfractionated heparin (UFH), low molecular weight heparins (LMWH) and ultra (U)LMWH (fondaparinux, as representative of synthetic pentasaccharides); modified from [29,31,32,33]. Abbreviations: AT: antithrombin; GAGs: glycosaminoglycans; h: hours; s.c.: subcutaneous; aPTT: activated partial thromboplastin time; ETP: endogenous thrombin potential; FXa: activated coagulation factor X; * in selected cases.

	UFH	LMWH	ULMWH
Molecular Weight (Da) (average)	3000–30,000 (19,000)	1000–10,000 (5000)	1728 -
Source	Isolation from porcine mucosa	Degradation from UFH	Chemical synthesis
Chemical characteristics	Highly variable mixture of GAGs	Highly variable mixture of GAGs	Chemically defined pentasaccharide
Mode of action	AT-mediated FII and FXa inhibition AT-independent effects	AT-mediated FII and FXa inhibition AT-independent effects	Selective AT-mediated FXa inhibition
Affinity to plasma proteins and cells	+++	+	No
Platelet interaction	+++	+	No
Bioavailability (s.c. [%])	10–30	85–98	100
Half-life time (s.c.)	1–4 h	3–5 h	17–21 h
Elimination	Renal, intestinal	Renal, intestinal	Renal
Therapy monitoring	aPTT, ETP, platelet count	* FXa, platelet count	No
Antagonist	Protamine	Protamine	No

## Data Availability

Not applicable.

## References

[1] Howell W.H. (1912). The nature and action of the thromboplastic (zymoplastic) substance of the tissues. Am. J. Physiol..

[2] McLean J. (1916). The Thromboplastic Action of Cephalin. Am. J. Physiol..

[3] Howell W.H., Holt E. (1918). Two New Factors in Blood Coagulation—Heparin and Pro- Antithrombin. Am. J. Physiol..

[4] Hemker H.C. (2016). A century of heparin: Past, present and future. J. Thromb. Haemost..

[5] Weitz J.I., Harenberg J. (2017). New developments in anticoagulants: Past, present and future. Thromb. Haemost..

[6] Dumaine R., Borentain M., Bertel O., Bode C., Gallo R., White H.D., Collet J.P., Steinhubl S.R., Montalescot G. (2007). Intravenous low-molecular-weight heparins compared with unfractionated heparin in percutaneous coronary intervention: Quantitative review of randomized trials. Arch Intern. Med..

[7] Sandercock P.A., Leong T.S. (2017). Low-molecular-weight heparins or heparinoids versus standard unfractionated heparin for acute ischaemic stroke. Cochrane Database Syst. Rev..

[8] Robertson L., Strachan J. (2017). Subcutaneous unfractionated heparin for the initial treatment of venous thromboembolism. Cochrane Database Syst. Rev..

[9] Schallmoser K., Bartmann C., Rohde E., Reinisch A., Kashofer K., Stadelmeyer E., Drexler C., Lanzer G., Linkesch W., Strunk D. (2007). Human platelet lysate can replace fetal bovine serum for clinical-scale expansion of functional mesenchymal stromal cells. Transfusion.

[10] Burnouf T., Strunk D., Koh M.B., Schallmoser K. (2016). Human platelet lysate: Replacing fetal bovine serum as a gold standard for human cell propagation?. Biomaterials.

[11] Trento C., Bernardo M.E., Nagler A., Kuci S., Bornhauser M., Kohl U., Strunk D., Galleu A., Sanchez-Guijo F., Gaipa G. (2018). Manufacturing Mesenchymal Stromal Cells for the Treatment of Graft-versus-Host Disease: A Survey among Centers Affiliated with the European Society for Blood and Marrow Transplantation. Biol. Blood Marrow Transpl..

[12] Liao L., Shi B., Chang H., Su X., Zhang L., Bi C., Shuai Y., Du X., Deng Z., Jin Y. (2017). Heparin improves BMSC cell therapy: Anticoagulant treatment by heparin improves the safety and therapeutic effect of bone marrow-derived mesenchymal stem cell cytotherapy. Theranostics.

[13] Moll G., Ankrum J.A., Kamhieh-Milz J., Bieback K., Ringden O., Volk H.D., Geissler S., Reinke P. (2019). Intravascular Mesenchymal Stromal/Stem Cell Therapy Product Diversification: Time for New Clinical Guidelines. Trends Mol. Med..

[14] Stephenne X., Nicastro E., Eeckhoudt S., Hermans C., Nyabi O., Lombard C., Najimi M., Sokal E. (2012). Bivalirudin in combination with heparin to control mesenchymal cell procoagulant activity. PLoS ONE.

[15] Chande N., McDonald J.W., Macdonald J.K., Wang J.J. (2010). Unfractionated or low-molecular weight heparin for induction of remission in ulcerative colitis. Cochrane Database Syst. Rev..

[16] Young E. (2008). The anti-inflammatory effects of heparin and related compounds. Thromb. Res..

[17] Montroy J., Lalu M.M., Auer R.C., Grigor E., Mazzarello S., Carrier M., Kimmelman J., Fergusson D.A. (2020). The Efficacy and Safety of Low Molecular Weight Heparin Administration to Improve Survival of Cancer Patients: A Systematic Review and Meta-Analysis. Thromb. Haemost..

[18] Garcia-Escobar I., Beato-Zambrano C., Munoz Langa J., Brozos Vazquez E., Obispo Portero B., Gutierrez-Abad D., Munoz Martin A.J., Cancer and Thrombosis Working Group of the Spanish Society of Medical Oncology (2018). Pleiotropic effects of heparins: Does anticoagulant treatment increase survival in cancer patients?. Clin. Transl. Oncol..

[19] Flumignan R.L., Tinoco J.D.S., Pascoal P.I., Areias L.L., Cossi M.S., Fernandes M.I., Costa I.K., Souza L., Matar C.F., Tendal B. (2020). Prophylactic anticoagulants for people hospitalised with COVID-19. Cochrane Database Syst. Rev..

[20] Scavone C., Mascolo A., Rafaniello C., Sportiello L., Trama U., Zoccoli A., Bernardi F.F., Racagni G., Berrino L., Castaldo G. (2021). Therapeutic strategies to fight COVID-19: Which is the status artis?. Br. J. Pharmacol.

[21] Casu B., Lindahl U. (2001). Structure and biological interactions of heparin and heparan sulfate. Adv. Carbohydr. Chem. Biochem..

[22] Shriver Z., Capila I., Venkataraman G., Sasisekharan R. (2012). Heparin and heparan sulfate: Analyzing structure and microheterogeneity. Handb. Exp. Pharmacol..

[23] Sarrazin S., Lamanna W.C., Esko J.D. (2011). Heparan sulfate proteoglycans. Cold Spring Harb. Perspect Biol..

[24] Da Silva E.Z., Jamur M.C., Oliver C. (2014). Mast cell function: A new vision of an old cell. J. Histochem. Cytochem..

[25] Forsberg E., Pejler G., Ringvall M., Lunderius C., Tomasini-Johansson B., Kusche-Gullberg M., Eriksson I., Ledin J., Hellman L., Kjellen L. (1999). Abnormal mast cells in mice deficient in a heparin-synthesizing enzyme. Nature.

[26] Humphries D.E., Wong G.W., Friend D.S., Gurish M.F., Qiu W.T., Huang C., Sharpe A.H., Stevens R.L. (1999). Heparin is essential for the storage of specific granule proteases in mast cells. Nature.

[27] Damus P.S., Hicks M., Rosenberg R.D. (1973). Anticoagulant action of heparin. Nature.

[28] Jordan R.E., Oosta G.M., Gardner W.T., Rosenberg R.D. (1980). The kinetics of hemostatic enzyme-antithrombin interactions in the presence of low molecular weight heparin. J. Biol. Chem..

[29] Baytas S.N., Linhardt R.J. (2020). Advances in the preparation and synthesis of heparin and related products. Drug Discov. Today.

[30] Szajek A.Y., Chess E., Johansen K., Gratzl G., Gray E., Keire D., Linhardt R.J., Liu J., Morris T., Mulloy B. (2016). The US regulatory and pharmacopeia response to the global heparin contamination crisis. Nat. Biotechnol..

[31] Alban S. (2005). From heparins to factor Xa inhibitors and beyond. Eur. J. Clin. Investig..

[32] Hemker H.C., Al Dieri R., Beguin S. (2019). Heparins: A Shift of Paradigm. Front. Med..

[33] Alban S., Pötzsch B., Madlener K. (2010). Hämostaseologie Grundlagen, Diagnostik, Therapie.

[34] Weitz J.I. (1997). Low-molecular-weight heparins. N. Engl. J. Med..

[35] Hirsh J., Warkentin T.E., Raschke R., Granger C., Ohman E.M., Dalen J.E. (1998). Heparin and low-molecular-weight heparin: Mechanisms of action, pharmacokinetics, dosing considerations, monitoring, efficacy, and safety. Chest.

[36] Chandarajoti K., Liu J., Pawlinski R. (2016). The design and synthesis of new synthetic low-molecular-weight heparins. J. Thromb. Haemost..

[37] Douketis J.D., Spyropoulos A.C., Spencer F.A., Mayr M., Jaffer A.K., Eckman M.H., Dunn A.S., Kunz R. (2012). Perioperative management of antithrombotic therapy: Antithrombotic Therapy and Prevention of Thrombosis, 9th ed: American College of Chest Physicians Evidence-Based Clinical Practice Guidelines. Chest.

[38] Thachil J., Tang N., Gando S., Falanga A., Cattaneo M., Levi M., Clark C., Iba T. (2020). ISTH interim guidance on recognition and management of coagulopathy in COVID-19. J. Thromb. Haemost..

[39] Swan D., Carrier M., Lisman T., Thachil J. (2021). Heparin—Messias or Verschlimmbesserung?. J. Thromb. Haemost..

[40] Barrett C.D., Moore H.B., Yaffe M.B., Moore E.E. (2020). ISTH interim guidance on recognition and management of coagulopathy in COVID-19: A Comment. J. Thromb. Haemost..

[41] Spyropoulos A.C., Goldin M., Giannis D., Diab W., Wang J., Khanijo S., Mignatti A., Gianos E., Cohen M., Sharifova G. (2021). Efficacy and Safety of Therapeutic-Dose Heparin vs Standard Prophylactic or Intermediate-Dose Heparins for Thromboprophylaxis in High-risk Hospitalized Patients With COVID-19: The HEP-COVID Randomized Clinical Trial. JAMA Intern. Med..

[42] Investigators I., Sadeghipour P., Talasaz A.H., Rashidi F., Sharif-Kashani B., Beigmohammadi M.T., Farrokhpour M., Sezavar S.H., Payandemehr P., Dabbagh A. (2021). Effect of Intermediate-Dose vs Standard-Dose Prophylactic Anticoagulation on Thrombotic Events, Extracorporeal Membrane Oxygenation Treatment, or Mortality Among Patients With COVID-19 Admitted to the Intensive Care Unit: The INSPIRATION Randomized Clinical Trial. JAMA.

[43] Lopes R.D., de Barros E.S.P.G.M., Furtado R.H.M., Macedo A.V.S., Bronhara B., Damiani L.P., Barbosa L.M., de Aveiro Morata J., Ramacciotti E., de Aquino Martins P. (2021). Therapeutic versus prophylactic anticoagulation for patients admitted to hospital with COVID-19 and elevated D-dimer concentration (ACTION): An open-label, multicentre, randomised, controlled trial. Lancet.

[44] (2021). COVID-19 Rapid Guideline: Managing COVID-19.

[45] Cuker A., Tseng E.K., Nieuwlaat R., Angchaisuksiri P., Blair C., Dane K., Davila J., DeSancho M.T., Diuguid D., Griffin D.O. (2021). American Society of Hematology 2021 guidelines on the use of anticoagulation for thromboprophylaxis in patients with COVID-19. Blood Adv..

[46] Lima M., Rudd T., Yates E. (2017). New Applications of Heparin and Other Glycosaminoglycans. Molecules.

[47] Harrop H.A., Coombe D.R., Rider C.C. (1994). Heparin specifically inhibits binding of V3 loop antibodies to HIV-1 gp120, an effect potentiated by CD4 binding. AIDS.

[48] Guryanov I., Real-Fernandez F., Sabatino G., Prisco N., Korzhikov-Vlakh V., Biondi B., Papini A.M., Korzhikova-Vlakh E., Rovero P., Tennikova T. (2019). Modeling interaction between gp120 HIV protein and CCR5 receptor. J. Pept. Sci..

[49] Marks R.M., Lu H., Sundaresan R., Toida T., Suzuki A., Imanari T., Hernaiz M.J., Linhardt R.J. (2001). Probing the interaction of dengue virus envelope protein with heparin: Assessment of glycosaminoglycan-derived inhibitors. J. Med. Chem..

[50] Rathore D., McCutchan T.F., Garboczi D.N., Toida T., Hernaiz M.J., LeBrun L.A., Lang S.C., Linhardt R.J. (2001). Direct measurement of the interactions of glycosaminoglycans and a heparin decasaccharide with the malaria circumsporozoite protein. Biochemistry.

[51] Tao L., Tian S., Zhang J., Liu Z., Robinson-McCarthy L., Miyashita S.I., Breault D.T., Gerhard R., Oottamasathien S., Whelan S.P.J. (2019). Sulfated glycosaminoglycans and low-density lipoprotein receptor contribute to Clostridium difficile toxin A entry into cells. Nat. Microbiol..

[52] Kim S.Y., Koetzner C.A., Payne A.F., Nierode G.J., Yu Y., Wang R., Barr E., Dordick J.S., Kramer L.D., Zhang F. (2019). Glycosaminoglycan Compositional Analysis of Relevant Tissues in Zika Virus Pathogenesis and in Vitro Evaluation of Heparin as an Antiviral against Zika Virus Infection. Biochemistry.

[53] Ludwig R.J. (2009). Therapeutic use of heparin beyond anticoagulation. Curr. Drug Discov. Technol..

[54] Mousavi S., Moradi M., Khorshidahmad T., Motamedi M. (2015). Anti-Inflammatory Effects of Heparin and Its Derivatives: A Systematic Review. Adv. Pharmacol. Sci..

[55] Streiff M.B. (2016). Thrombosis in the setting of cancer. Hematol. Am. Soc. Hematol. Educ. Program..

[56] Hisada Y., Mackman N. (2017). Cancer-associated pathways and biomarkers of venous thrombosis. Blood.

[57] Simanek R., Vormittag R., Ay C., Alguel G., Dunkler D., Schwarzinger I., Steger G., Jaeger U., Zielinski C., Pabinger I. (2010). High platelet count associated with venous thromboembolism in cancer patients: Results from the Vienna Cancer and Thrombosis Study (CATS). J. Thromb. Haemost..

[58] Lyman G.H., Carrier M., Ay C., Di Nisio M., Hicks L.K., Khorana A.A., Leavitt A.D., Lee A.Y.Y., Macbeth F., Morgan R.L. (2021). American Society of Hematology 2021 guidelines for management of venous thromboembolism: Prevention and treatment in patients with cancer. Blood Adv..

[59] Falanga A., Gal G.L., Carrier M., Abdel-Razeq H., Ay C., Martin A.J.M., Rocha A.T.C., Agnelli G., Elalamy I., Brenner B. (2021). Management of Cancer-Associated Thrombosis: Unmet Needs and Future Perspectives. TH Open.

[60] Haemmerle M., Stone R.L., Menter D.G., Afshar-Kharghan V., Sood A.K. (2018). The Platelet Lifeline to Cancer: Challenges and Opportunities. Cancer Cell.

[61] Lee E.C., Cameron S.J. (2017). Cancer and Thrombotic Risk: The Platelet Paradigm. Front. Cardiovasc. Med..

[62] Salzman E.W., Rosenberg R.D., Smith M.H., Lindon J.N., Favreau L. (1980). Effect of heparin and heparin fractions on platelet aggregation. J. Clin. Investig..

[63] Koenig A., Norgard-Sumnicht K., Linhardt R., Varki A. (1998). Differential interactions of heparin and heparan sulfate glycosaminoglycans with the selectins. Implications for the use of unfractionated and low molecular weight heparins as therapeutic agents. J. Clin. Investig..

[64] Borsig L., Wong R., Feramisco J., Nadeau D.R., Varki N.M., Varki A. (2001). Heparin and cancer revisited: Mechanistic connections involving platelets, P-selectin, carcinoma mucins, and tumor metastasis. Proc. Natl. Acad. Sci. USA.

[65] Lippman M. (1957). The growth-inhibitory action of heparin on the Ehrlich ascites tumor in mice. Cancer Res..

[66] Noble S. (2014). Heparins and cancer survival: Where do we stand?. Thromb. Res..

[67] Ripsman D., Fergusson D.A., Montroy J., Auer R.C., Huang J.W., Dobriyal A., Wesch N., Carrier M., Lalu M.M. (2020). A systematic review on the efficacy and safety of low molecular weight heparin as an anticancer therapeutic in preclinical animal models. Thromb. Res..

[68] Mohamed S., Coombe D.R. (2017). Heparin Mimetics: Their Therapeutic Potential. Pharmaceuticals.

[69] Dhakal P., Rayamajhi S., Verma V., Gundabolu K., Bhatt V.R. (2017). Reversal of Anticoagulation and Management of Bleeding in Patients on Anticoagulants. Clin. Appl. Thromb. Hemost..

[70] Weismann R.E., Tobin R.W. (1958). Arterial embolism occurring during systemic heparin therapy. AMA Arch Surg..

[71] Arepally G.M. (2017). Heparin-induced thrombocytopenia. Blood.

[72] Cuker A., Arepally G.M., Chong B.H., Cines D.B., Greinacher A., Gruel Y., Linkins L.A., Rodner S.B., Selleng S., Warkentin T.E. (2018). American Society of Hematology 2018 guidelines for management of venous thromboembolism: Heparin-induced thrombocytopenia. Blood Adv..

[73] Onishi A., St Ange K., Dordick J.S., Linhardt R.J. (2016). Heparin and anticoagulation. Front. Biosci..

[74] Mouw J.K., Ou G., Weaver V.M. (2014). Extracellular matrix assembly: A multiscale deconstruction. Nat. Rev. Mol. Cell Biol..

[75] Singh P., Carraher C., Schwarzbauer J.E. (2010). Assembly of fibronectin extracellular matrix. Annu. Rev. Cell Dev. Biol..

[76] Hubbard B., Buczek-Thomas J.A., Nugent M.A., Smith M.L. (2014). Heparin-dependent regulation of fibronectin matrix conformation. Matrix Biol..

[77] Cole G.J., Loewy A., Glaser L. (1986). Neuronal cell-cell adhesion depends on interactions of N-CAM with heparin-like molecules. Nature.

[78] Peysselon F., Ricard-Blum S. (2014). Heparin-protein interactions: From affinity and kinetics to biological roles. Application to an interaction network regulating angiogenesis. Matrix Biol..

[79] Medeiros V.P., Paredes-Gamero E.J., Monteiro H.P., Rocha H.A., Trindade E.S., Nader H.B. (2012). Heparin-integrin interaction in endothelial cells: Downstream signaling and heparan sulfate expression. J. Cell Physiol..

[80] Peter K., Schwarz M., Conradt C., Nordt T., Moser M., Kubler W., Bode C. (1999). Heparin inhibits ligand binding to the leukocyte integrin Mac-1 (CD11b/CD18). Circulation.

[81] Helledie T., Dombrowski C., Rai B., Lim Z.X., Hin I.L., Rider D.A., Stein G.S., Hong W., van Wijnen A.J., Hui J.H. (2012). Heparan sulfate enhances the self-renewal and therapeutic potential of mesenchymal stem cells from human adult bone marrow. Stem Cells Dev..

[82] Ling L., Camilleri E.T., Helledie T., Samsonraj R.M., Titmarsh D.M., Chua R.J., Dreesen O., Dombrowski C., Rider D.A., Galindo M. (2016). Effect of heparin on the biological properties and molecular signature of human mesenchymal stem cells. Gene.

[83] Laner-Plamberger S., Oeller M., Poupardin R., Krisch L., Hochmann S., Kalathur R., Pachler K., Kreutzer C., Erdmann G., Rohde E. (2019). Heparin Differentially Impacts Gene Expression of Stromal Cells from Various Tissues. Sci. Rep..

[84] Schlessinger J., Plotnikov A.N., Ibrahimi O.A., Eliseenkova A.V., Yeh B.K., Yayon A., Linhardt R.J., Mohammadi M. (2000). Crystal structure of a ternary FGF-FGFR-heparin complex reveals a dual role for heparin in FGFR binding and dimerization. Mol. Cell.

[85] Pellegrini L., Burke D.F., von Delft F., Mulloy B., Blundell T.L. (2000). Crystal structure of fibroblast growth factor receptor ectodomain bound to ligand and heparin. Nature.

[86] Dudas J., Ramadori G., Knittel T., Neubauer K., Raddatz D., Egedy K., Kovalszky I. (2000). Effect of heparin and liver heparan sulphate on interaction of HepG2-derived transcription factors and their cis-acting elements: Altered potential of hepatocellular carcinoma heparan sulphate. Biochem J..

[87] Pandey M.S., Weigel P.H. (2014). Hyaluronic acid receptor for endocytosis (HARE)-mediated endocytosis of hyaluronan, heparin, dermatan sulfate, and acetylated low density lipoprotein (AcLDL), but not chondroitin sulfate types A, C, D, or E, activates NF-kappaB-regulated gene expression. J. Biol. Chem..

[88] Pandey M.S., Miller C.M., Harris E.N., Weigel P.H. (2016). Activation of ERK and NF-kappaB during HARE-Mediated Heparin Uptake Require Only One of the Four Endocytic Motifs. PLoS ONE.

[89] Harris E.N., Weigel J.A., Weigel P.H. (2008). The human hyaluronan receptor for endocytosis (HARE/Stabilin-2) is a systemic clearance receptor for heparin. J. Biol. Chem..

[90] Jaffe E.A., Nachman R.L., Becker C.G., Minick C.R. (1973). Culture of human endothelial cells derived from umbilical veins. Identification by morphologic and immunologic criteria. J. Clin. Investig..

[91] Spivak-Kroizman T., Lemmon M.A., Dikic I., Ladbury J.E., Pinchasi D., Huang J., Jaye M., Crumley G., Schlessinger J., Lax I. (1994). Heparin-induced oligomerization of FGF molecules is responsible for FGF receptor dimerization, activation, and cell proliferation. Cell.

[92] Rider C.C., Mulloy B. (2017). Heparin, Heparan Sulphate and the TGF-beta Cytokine Superfamily. Molecules.

[93] Lee J., Wee S., Gunaratne J., Chua R.J., Smith R.A., Ling L., Fernig D.G., Swaminathan K., Nurcombe V., Cool S.M. (2015). Structural determinants of heparin-transforming growth factor-beta1 interactions and their effects on signaling. Glycobiology.

[94] Krilleke D., DeErkenez A., Schubert W., Giri I., Robinson G.S., Ng Y.S., Shima D.T. (2007). Molecular mapping and functional characterization of the VEGF164 heparin-binding domain. J. Biol. Chem..

[95] Jeong K.W., Jeong M.C., Jin B., Kim Y. (2013). Relationship between structural flexibility and function in the C-terminal region of the heparin-binding domain of VEGF165. Biochemistry.

[96] De Falco S. (2012). The discovery of placenta growth factor and its biological activity. Exp. Mol. Med..

[97] Yoo S.A., Kim M., Kang M.C., Kong J.S., Kim K.M., Lee S., Hong B.K., Jeong G.H., Lee J., Shin M.G. (2019). Placental growth factor regulates the generation of TH17 cells to link angiogenesis with autoimmunity. Nat. Immunol..

[98] Rolny C., Spillmann D., Lindahl U., Claesson-Welsh L. (2002). Heparin amplifies platelet-derived growth factor (PDGF)- BB-induced PDGF alpha -receptor but not PDGF beta -receptor tyrosine phosphorylation in heparan sulfate-deficient cells. Effects on signal transduction and biological responses. J. Biol. Chem..

[99] Zhou F.Y., Owens R.T., Hermonen J., Jalkanen M., Hook M. (1997). Is the sensitivity of cells for FGF-1 and FGF-2 regulated by cell surface heparan sulfate proteoglycans?. Eur. J. Cell Biol..

[100] Allen B.L., Filla M.S., Rapraeger A.C. (2001). Role of heparan sulfate as a tissue-specific regulator of FGF-4 and FGF receptor recognition. J. Cell Biol..

[101] DiGabriele A.D., Lax I., Chen D.I., Svahn C.M., Jaye M., Schlessinger J., Hendrickson W.A. (1998). Structure of a heparin-linked biologically active dimer of fibroblast growth factor. Nature.

[102] Kadomatsu K., Kishida S., Tsubota S. (2013). The heparin-binding growth factor midkine: The biological activities and candidate receptors. J. Biochem..

[103] Sakata H., Stahl S.J., Taylor W.G., Rosenberg J.M., Sakaguchi K., Wingfield P.T., Rubin J.S. (1997). Heparin binding and oligomerization of hepatocyte growth factor/scatter factor isoforms. Heparan sulfate glycosaminoglycan requirement for Met binding and signaling. J. Biol. Chem..

[104] Capila I., Linhardt R.J. (2002). Heparin-protein interactions. Angew. Chem. Int. Ed. Engl..

[105] Saksela O., Moscatelli D., Sommer A., Rifkin D.B. (1988). Endothelial cell-derived heparan sulfate binds basic fibroblast growth factor and protects it from proteolytic degradation. J. Cell Biol..

[106] Gospodarowicz D., Cheng J. (1986). Heparin protects basic and acidic FGF from inactivation. J. Cell Physiol..

[107] Flaumenhaft R., Moscatelli D., Rifkin D.B. (1990). Heparin and heparan sulfate increase the radius of diffusion and action of basic fibroblast growth factor. J. Cell Biol..

[108] Maciag T., Mehlman T., Friesel R., Schreiber A.B. (1984). Heparin binds endothelial cell growth factor, the principal endothelial cell mitogen in bovine brain. Science.

[109] Thornton S.C., Mueller S.N., Levine E.M. (1983). Human endothelial cells: Use of heparin in cloning and long-term serial cultivation. Science.

[110] Laner-Plamberger S., Oeller M., Mrazek C., Hartl A., Sonderegger A., Rohde E., Strunk D., Schallmoser K. (2019). Upregulation of mitotic bookmarking factors during enhanced proliferation of human stromal cells in human platelet lysate. J. Transl. Med..

[111] Furue M.K., Na J., Jackson J.P., Okamoto T., Jones M., Baker D., Hata R., Moore H.D., Sato J.D., Andrews P.W. (2008). Heparin promotes the growth of human embryonic stem cells in a defined serum-free medium. Proc. Natl. Acad. Sci. USA.

[112] Hemeda H., Kalz J., Walenda G., Lohmann M., Wagner W. (2013). Heparin concentration is critical for cell culture with human platelet lysate. Cytotherapy.

[113] Hacker U., Nybakken K., Perrimon N. (2005). Heparan sulphate proteoglycans: The sweet side of development. Nat. Rev. Mol. Cell Biol..

[114] Holley R.J., Meade K.A., Merry C.L. (2014). Using embryonic stem cells to understand how glycosaminoglycans regulate differentiation. Biochem. Soc. Trans..

[115] Mathews S., Mathew S.A., Gupta P.K., Bhonde R., Totey S. (2014). Glycosaminoglycans enhance osteoblast differentiation of bone marrow derived human mesenchymal stem cells. J. Tissue Eng. Regen. Med..

[116] Simann M., Schneider V., Le Blanc S., Dotterweich J., Zehe V., Krug M., Jakob F., Schilling T., Schutze N. (2015). Heparin affects human bone marrow stromal cell fate: Promoting osteogenic and reducing adipogenic differentiation and conversion. Bone.

[117] Li B., Lin Z., Mitsi M., Zhang Y., Vogel V. (2015). Heparin-induced conformational changes of fibronectin within the extracellular matrix promote hMSC osteogenic differentiation. Biomater. Sci..

[118] Kim J.M., Lee K., Kim M.Y., Shin H.I., Jeong D. (2018). Suppressive effect of syndecan ectodomains and N-desulfated heparins on osteoclastogenesis via direct binding to macrophage-colony stimulating factor. Cell Death Dis..

[119] Baud’huin M., Ruiz-Velasco C., Jego G., Charrier C., Gasiunas N., Gallagher J., Maillasson M., Naggi A., Padrines M., Redini F. (2011). Glycosaminoglycans inhibit the adherence and the spreading of osteoclasts and their precursors: Role in osteoclastogenesis and bone resorption. Eur. J. Cell Biol..

[120] Griffith G.C., Nichols G., Asher J.D., Flanagan B. (1965). Heparin Osteoporosis. JAMA.

[121] Barbour L.A., Kick S.D., Steiner J.F., LoVerde M.E., Heddleston L.N., Lear J.L., Baron A.E., Barton P.L. (1994). A prospective study of heparin-induced osteoporosis in pregnancy using bone densitometry. Am. J. Obstet. Gynecol..

[122] Gajic-Veljanoski O., Phua C.W., Shah P.S., Cheung A.M. (2016). Effects of Long-Term Low-Molecular-Weight Heparin on Fractures and Bone Density in Non-Pregnant Adults: A Systematic Review with Meta-Analysis. J. Gen. Intern. Med..

[123] Gimbrone M.A., Cotran R.S., Folkman J. (1974). Human vascular endothelial cells in culture. Growth and DNA synthesis. J. Cell Biol..

[124] Reinisch A., Hofmann N.A., Obenauf A.C., Kashofer K., Rohde E., Schallmoser K., Flicker K., Lanzer G., Linkesch W., Speicher M.R. (2009). Humanized large-scale expanded endothelial colony-forming cells function in vitro and in vivo. Blood.

[125] Reinisch A., Etchart N., Thomas D., Hofmann N.A., Fruehwirth M., Sinha S., Chan C.K., Senarath-Yapa K., Seo E.Y., Wearda T. (2015). Epigenetic and in vivo comparison of diverse MSC sources reveals an endochondral signature for human hematopoietic niche formation. Blood.

[126] EMA (2007). Guideline on Human Cell-Based Medicinal Products. Off. J. Eur. Union..

[127] Doucet C., Ernou I., Zhang Y., Llense J.R., Begot L., Holy X., Lataillade J.J. (2005). Platelet lysates promote mesenchymal stem cell expansion: A safety substitute for animal serum in cell-based therapy applications. J. Cell Physiol..

[128] Schallmoser K., Rohde E., Bartmann C., Obenauf A.C., Reinisch A., Strunk D. (2009). Platelet-derived growth factors for GMP-compliant propagation of mesenchymal stromal cells. Biomed Mater Eng..

[129] Ansari M., Strunk D., Schallmoser K., Delco C., Rougemont A.L., Moll S., Villard J., Gumy-Pause F., Chalandon Y., Parvex P. (2012). Third-party mesenchymal stromal cell infusion is associated with a decrease in thrombotic microangiopathy symptoms observed post-hematopoietic stem cell transplantation. Pediatr. Transpl..

[130] Warnke P.H., Humpe A., Strunk D., Stephens S., Warnke F., Wiltfang J., Schallmoser K., Alamein M., Bourke R., Heiner P. (2013). A clinically-feasible protocol for using human platelet lysate and mesenchymal stem cells in regenerative therapies. J. Craniomaxillofac Surg..

[131] Bartmann C., Rohde E., Schallmoser K., Purstner P., Lanzer G., Linkesch W., Strunk D. (2007). Two steps to functional mesenchymal stromal cells for clinical application. Transfusion.

[132] Bieback K., Hecker A., Kocaomer A., Lannert H., Schallmoser K., Strunk D., Kluter H. (2009). Human alternatives to fetal bovine serum for the expansion of mesenchymal stromal cells from bone marrow. Stem Cells.

[133] Hemeda H., Giebel B., Wagner W. (2014). Evaluation of human platelet lysate versus fetal bovine serum for culture of mesenchymal stromal cells. Cytotherapy.

[134] Bieback K., Fernandez-Munoz B., Pati S., Schafer R. (2019). Gaps in the knowledge of human platelet lysate as a cell culture supplement for cell therapy: A joint publication from the AABB and the International Society of Cell Therapy. Cytotherapy.

[135] Lord M.S., Jung M., Whitelock J.M. (2017). Optimization of bioengineered heparin/heparan sulfate production for therapeutic applications. Bioengineered.

[136] Kelton J.G., Arnold D.M., Bates S.M. (2013). Nonheparin anticoagulants for heparin-induced thrombocytopenia. N. Engl. J. Med..

[137] Gustafson E.K., Elgue G., Hughes R.D., Mitry R.R., Sanchez J., Haglund U., Meurling S., Dhawan A., Korsgren O., Nilsson B. (2011). The instant blood-mediated inflammatory reaction characterized in hepatocyte transplantation. Transplantation.

[138] Galipeau J., Sensebe L. (2018). Mesenchymal Stromal Cells: Clinical Challenges and Therapeutic Opportunities. Cell Stem Cell.

[139] Bennet W., Groth C.G., Larsson R., Nilsson B., Korsgren O. (2000). Isolated human islets trigger an instant blood mediated inflammatory reaction: Implications for intraportal islet transplantation as a treatment for patients with type 1 diabetes. Ups J. Med. Sci..

[140] Bennet W., Sundberg B., Groth C.G., Brendel M.D., Brandhorst D., Brandhorst H., Bretzel R.G., Elgue G., Larsson R., Nilsson B. (1999). Incompatibility between human blood and isolated islets of Langerhans: A finding with implications for clinical intraportal islet transplantation?. Diabetes.

[141] Quimby J.M., Webb T.L., Habenicht L.M., Dow S.W. (2013). Safety and efficacy of intravenous infusion of allogeneic cryopreserved mesenchymal stem cells for treatment of chronic kidney disease in cats: Results of three sequential pilot studies. Stem Cell Res. Ther..

[142] Gleeson B.M., Martin K., Ali M.T., Kumar A.H., Pillai M.G., Kumar S.P., O’Sullivan J.F., Whelan D., Stocca A., Khider W. (2015). Bone Marrow-Derived Mesenchymal Stem Cells Have Innate Procoagulant Activity and Cause Microvascular Obstruction Following Intracoronary Delivery: Amelioration by Antithrombin Therapy. Stem. Cells.

[143] Moll G., Geissler S., Catar R., Ignatowicz L., Hoogduijn M.J., Strunk D., Bieback K., Ringden O. (2016). Cryopreserved or Fresh Mesenchymal Stromal Cells: Only a Matter of Taste or Key to Unleash the Full Clinical Potential of MSC Therapy?. Adv. Exp. Med. Biol..

[144] Moll G., Rasmusson-Duprez I., von Bahr L., Connolly-Andersen A.M., Elgue G., Funke L., Hamad O.A., Lonnies H., Magnusson P.U., Sanchez J. (2012). Are therapeutic human mesenchymal stromal cells compatible with human blood?. Stem Cells.

[145] Oeller M., Laner-Plamberger S., Hochmann S., Ketterl N., Feichtner M., Brachtl G., Hochreiter A., Scharler C., Bieler L., Romanelli P. (2018). Selection of Tissue Factor-Deficient Cell Transplants as a Novel Strategy for Improving Hemocompatibility of Human Bone Marrow Stromal Cells. Theranostics.

[146] Tatsumi K., Ohashi K., Matsubara Y., Kohori A., Ohno T., Kakidachi H., Horii A., Kanegae K., Utoh R., Iwata T. (2013). Tissue factor triggers procoagulation in transplanted mesenchymal stem cells leading to thromboembolism. Biochem. Biophys. Res. Commun..

[147] Sadeghi B., Moretti G., Arnberg F., Samen E., Kohein B., Catar R., Kamhieh-Milz J., Geissler S., Moll G., Holmin S. (2019). Preclinical Toxicity Evaluation of Clinical Grade Placenta-Derived Decidua Stromal Cells. Front. Immunol..

[148] Baccarani U., Adani G.L., Sanna A., Avellini C., Sainz-Barriga M., Lorenzin D., Montanaro D., Gasparini D., Risaliti A., Donini A. (2005). Portal vein thrombosis after intraportal hepatocytes transplantation in a liver transplant recipient. Transpl. Int..

[149] Jonsson T.B., Larzon T., Arfvidsson B., Tidefelt U., Axelsson C.G., Jurstrand M., Norgren L. (2012). Adverse events during treatment of critical limb ischemia with autologous peripheral blood mononuclear cell implant. Int. Angiol..

[150] Tseng J., Citrin D.E., Waldman M., White D.E., Rosenberg S.A., Yang J.C. (2014). Thrombotic microangiopathy in metastatic melanoma patients treated with adoptive cell therapy and total body irradiation. Cancer.

[151] Cabric S., Sanchez J., Lundgren T., Foss A., Felldin M., Kallen R., Salmela K., Tibell A., Tufveson G., Larsson R. (2007). Islet surface heparinization prevents the instant blood-mediated inflammatory reaction in islet transplantation. Diabetes.

[152] Song G., Hu Y., Liu Y., Jiang R. (2018). Layer-by-Layer Heparinization of the Cell Surface by Using Heparin-Binding Peptide Functionalized Human Serum Albumin. Materials.

[153] Coppin L.C.F., Smets F., Ambroise J., Sokal E.E.M., Stephenne X. (2020). Infusion-related thrombogenesis by liver-derived mesenchymal stem cells controlled by anticoagulant drugs in 11 patients with liver-based metabolic disorders. Stem Cell Res. Ther..

[154] Coppin L., Najimi M., Bodart J., Rouchon M.S., van der Smissen P., Eeckhoudt S., Dahlqvist G., Castanares-Zapatero D., Komuta M., Brouns S.L. (2019). Clinical Protocol to Prevent Thrombogenic Effect of Liver-Derived Mesenchymal Cells for Cell-Based Therapies. Cells.

[155] Asif S., Ekdahl K.N., Fromell K., Gustafson E., Barbu A., Le Blanc K., Nilsson B., Teramura Y. (2016). Heparinization of cell surfaces with short peptide-conjugated PEG-lipid regulates thromboinflammation in transplantation of human MSCs and hepatocytes. Acta Biomater..

[156] Askari A.T., Unzek S., Popovic Z.B., Goldman C.K., Forudi F., Kiedrowski M., Rovner A., Ellis S.G., Thomas J.D., DiCorleto P.E. (2003). Effect of stromal-cell-derived factor 1 on stem-cell homing and tissue regeneration in ischaemic cardiomyopathy. Lancet.

[157] Hachim D., Whittaker T.E., Kim H., Stevens M.M. (2019). Glycosaminoglycan-based biomaterials for growth factor and cytokine delivery: Making the right choices. J. Control. Release.

[158] Yi H., Forsythe S., He Y., Liu Q., Xiong G., Wei S., Li G., Atala A., Skardal A., Zhang Y. (2017). Tissue-specific extracellular matrix promotes myogenic differentiation of human muscle progenitor cells on gelatin and heparin conjugated alginate hydrogels. Acta Biomater..

[159] Park D.S.J., Mewhort H.E.M., Teng G., Belke D., Turnbull J., Svystonyuk D., Guzzardi D., Kang S., Fedak P.W.M. (2018). Heparin Augmentation Enhances Bioactive Properties of Acellular Extracellular Matrix Scaffold. Tissue Eng. Part A.

[160] Qazi T.H., Mooney D.J., Pumberger M., Geissler S., Duda G.N. (2015). Biomaterials based strategies for skeletal muscle tissue engineering: Existing technologies and future trends. Biomaterials.

[161] Pumberger M., Qazi T.H., Ehrentraut M.C., Textor M., Kueper J., Stoltenburg-Didinger G., Winkler T., von Roth P., Reinke S., Borselli C. (2016). Synthetic niche to modulate regenerative potential of MSCs and enhance skeletal muscle regeneration. Biomaterials.

[162] Su D., Zhou J., Ahmed K.S., Ma Q., Lv G., Chen J. (2019). Fabrication and characterization of collagen-heparin-polypyrrole composite conductive film for neural scaffold. Int. J. Biol. Macromol..

[163] Newland B., Ehret F., Hoppe F., Eigel D., Pette D., Newland H., Welzel P.B., Kempermann G., Werner C. (2020). Macroporous heparin-based microcarriers allow long-term 3D culture and differentiation of neural precursor cells. Biomaterials.

[164] Aleahmad F., Ebrahimi S., Salmannezhad M., Azarnia M., Jaberipour M., Hoseini M., Talaei-Khozani T. (2017). Heparin/Collagen 3D Scaffold Accelerates Hepatocyte Differentiation of Wharton’s Jelly-Derived Mesenchymal Stem Cells. Tissue Eng. Regen Med..

[165] Qazi T.H., Mooney D.J., Duda G.N., Geissler S. (2017). Biomaterials that promote cell-cell interactions enhance the paracrine function of MSCs. Biomaterials.

[166] Chu H., Gao J., Chen C.W., Huard J., Wang Y. (2011). Injectable fibroblast growth factor-2 coacervate for persistent angiogenesis. Proc. Natl. Acad. Sci. USA.

[167] Wan X., Li P., Jin X., Su F., Shen J., Yuan J. (2020). Poly(epsilon-caprolactone)/keratin/heparin/VEGF biocomposite mats for vascular tissue engineering. J. Biomed Mater. Res. A.

[168] Tan J., Cui Y., Zeng Z., Wei L., Li L., Wang H., Hu H., Liu T., Huang N., Chen J. (2020). Heparin/poly-l-lysine nanoplatform with growth factor delivery for surface modification of cardiovascular stents: The influence of vascular endothelial growth factor loading. J. Biomed Mater Res. A.

[169] Lee K.I., Olmer M., Baek J., D’Lima D.D., Lotz M.K. (2018). Platelet-derived growth factor-coated decellularized meniscus scaffold for integrative healing of meniscus tears. Acta Biomater..

[170] Lee J., Yoo J.J., Atala A., Lee S.J. (2012). The effect of controlled release of PDGF-BB from heparin-conjugated electrospun PCL/gelatin scaffolds on cellular bioactivity and infiltration. Biomaterials.

[171] Ao Q., Wang S., He Q., Ten H., Oyama K., Ito A., He J., Javed R., Wang A., Matsuno A. (2020). Fibrin Glue/Fibronectin/Heparin-Based Delivery System of BMP2 Induces Osteogenesis in MC3T3-E1 Cells and Bone Formation in Rat Calvarial Critical-Sized Defects. ACS Appl. Mater Interfaces.

[172] Awada H.K., Long D.W., Wang Z., Hwang M.P., Kim K., Wang Y. (2017). A single injection of protein-loaded coacervate-gel significantly improves cardiac function post infarction. Biomaterials.

[173] Chen W.C., Lee B.G., Park D.W., Kim K., Chu H., Kim K., Huard J., Wang Y. (2015). Controlled dual delivery of fibroblast growth factor-2 and Interleukin-10 by heparin-based coacervate synergistically enhances ischemic heart repair. Biomaterials.

[174] Mays E.A., Kallakuri S.S., Sundararaghavan H.G. (2020). Heparin-hyaluronic acid nanofibers for growth factor sequestration in spinal cord repair. J. Biomed Mater Res. A.

[175] Liu G., Wu R., Yang B., Shi Y., Deng C., Atala A., Mou S., Criswell T., Zhang Y. (2020). A cocktail of growth factors released from a heparin hyaluronic-acid hydrogel promotes the myogenic potential of human urine-derived stem cells in vivo. Acta Biomater..

[176] Nillesen S.T., Geutjes P.J., Wismans R., Schalkwijk J., Daamen W.F., van Kuppevelt T.H. (2007). Increased angiogenesis and blood vessel maturation in acellular collagen-heparin scaffolds containing both FGF2 and VEGF. Biomaterials.

[177] Skop N.B., Calderon F., Cho C.H., Gandhi C.D., Levison S.W. (2016). Optimizing a multifunctional microsphere scaffold to improve neural precursor cell transplantation for traumatic brain injury repair. J. Tissue Eng. Regen Med..

[178] Park K.M., Son J.Y., Choi J.H., Kim I.G., Lee Y., Lee J.Y., Park K.D. (2014). Macro/Nano-gel composite as an injectable and bioactive bulking material for the treatment of urinary incontinence. Biomacromolecules.

[179] Hettiaratchi M.H., Krishnan L., Rouse T., Chou C., McDevitt T.C., Guldberg R.E. (2020). Heparin-mediated delivery of bone morphogenetic protein-2 improves spatial localization of bone regeneration. Sci. Adv..

[180] Wu J., Zhu J., He C., Xiao Z., Ye J., Li Y., Chen A., Zhang H., Li X., Lin L. (2016). Comparative Study of Heparin-Poloxamer Hydrogel Modified bFGF and aFGF for in Vivo Wound Healing Efficiency. ACS Appl. Mater. Interfaces.

[181] Niu G., Choi J.S., Wang Z., Skardal A., Giegengack M., Soker S. (2014). Heparin-modified gelatin scaffolds for human corneal endothelial cell transplantation. Biomaterials.

